# An update on inflammatory choroidal neovascularization: epidemiology, multimodal imaging, and management

**DOI:** 10.1186/s12348-018-0155-6

**Published:** 2018-09-12

**Authors:** Aniruddha Agarwal, Alessandro Invernizzi, Rohan Bir Singh, William Foulsham, Kanika Aggarwal, Sabia Handa, Rupesh Agrawal, Carlos Pavesio, Vishali Gupta

**Affiliations:** 1 Advanced Eye Center, Department of Ophthalmology, Postgraduate Institute of Medical Education and Research (PGIMER), Sector 12, Chandigarh, 160012 India; 2 Eye Clinic, Department of Biomedical and Clinical Science “L. Sacco”, Luigi Sacco Hospital, University of Milan, Milan, Italy; 3 Schepens Eye Research Institute, Massachusetts Eye and Ear Infirmary, Harvard Medical School, Boston, MA USA; 4 National Healthcare Group Eye Institute, Tan Tock Seng Hospital, Singapore, Singapore; 5 Moorfields Eye Hospital, NHS Foundation Trust, London, UK; 6 Singapore Eye Research Institute, Singapore, Singapore

**Keywords:** Inflammatory choroidal neovascularization, Uveitis, Posterior uveitis, Choroiditis, Indocyanine green angiography, Fluorescein angiography, Optical coherence tomography angiography, EDI-OCT

## Abstract

Inflammatory choroidal neovascular membranes are challenging to diagnose and manage. A number of uveitic entities may be complicated by the development of choroidal neovascularization leading to a decrease in central visual acuity. In conditions such as punctate inner choroidopathy, development of choroidal neovascularization is extremely common and must be suspected in all cases. On the other hand, in patients with conditions such as serpiginous choroiditis, and multifocal choroiditis, it may be difficult to differentiate between inflammatory choroiditis lesions and choroidal neovascularization. Multimodal imaging analysis, including the recently introduced technology of optical coherence tomography angiography, greatly aid in the diagnosis and management of inflammatory choroidal neovascularization. Management of these neovascular membranes consists of anti-vascular growth factor agents, with or without concomitant anti-inflammatory and/or corticosteroid therapy.

## Review

### Introduction

Choroidal neovascular membranes (CNV) represent the pathological growth of blood vessels and can result in loss of visual function. A diverse array of pathological processes involving the retinal pigment epithelium (RPE) and Bruch’s membrane may lead to the formation of CNV. Age-related macular degeneration (AMD) and myopia are the conditions that most commonly lead to the development of CNV, with ocular inflammation being the next most frequently implicated [1–3]. CNV may occur in a wide range of uveitides including both infectious and non-infectious etiologies. Notably, the incidence of CNV in posterior uveitis varies considerably depending upon the underlying pathological mechanism. For instance, the development of CNV is intricately connected to the morbidity associated with punctate inner choroidopathy (PIC) [4]. Other conditions that can be complicated by the development of CNV include multifocal choroiditis, serpiginous choroiditis, presumed ocular histoplasmosis syndrome (POHS), toxoplasma retinochoroiditis, and Vogt-Koyanagi-Harada (VKH) disease [5].

Inflammatory CNV (i-CNV) can occur either directly from an angiogenic stimulus mediated by local inflammation, result from a degenerative disruption in the Bruch’s membrane–RPE complex, or both [2, 6]. Neovascular buds grow through the damaged RPE–Bruch’s complex and proliferate to develop large branching vascular networks. These vessels progressively grow and leak, leading to the accumulation of fluid in the subretinal space.

The diagnosis of CNV in inflammatory eye conditions was historically challenging. Recently, the ocular imaging tools by which clinicians diagnose and manage CNV have undergone significant advances. For example, the development of optical coherence tomography angiography (OCTA) provides a highly valuable instrument to monitor the progression of CNV. Furthermore, there has been a rapid expansion in the available treatment modalities for i-CNV. In the past, laser photocoagulation and surgical excision were the only techniques employed in the management of i-CNVs. Treatment with intravitreal anti-vascular endothelial growth factor (VEGF) injections are now the mainstream in the management of i-CNVs. Other therapeutic strategies include photodynamic therapy (PDT) and corticosteroids/immunosuppressive agents (local and/or systemic) [1].

In this index review, a comprehensive overview of the epidemiology, clinical features, imaging characteristics, and management options of inflammatory choroidal neovascular membranes has been performed.

### Epidemiology of inflammatory CNV

CNV is an important sequela of a wide range of ophthalmic pathologies. The most common cause of CNV in the elderly is age-related macular degeneration, while in the young, CNV is frequently identified as secondary to high myopia, hereditary disorders, angioid streaks, and inflammation [7, 8]. Given the propensity of untreated CNV to result in rapid irreversible central vision loss, the importance of characterizing risk factors for CNV and prompt diagnosis is well-recognized [5]. Indeed, central vision loss due to CNV compromises patients’ ability to participate in certain types of work, as well as other daily activities such as reading and driving [9]. Uveitis tends to affect a working age population, and thus i-CNV frequently afflicts patients during their most productive and active years [5].

Various causes of infectious and non-infectious i-CNVs have been listed in Table 1 [10].

**Table 1:** Various uveitic entities associated with inflammatory choroidal neovascular membranes

Non-infectious	Choroiditis	Multifocal choroiditis
		Punctate inner choroidopathy
		Acute multifocal placoid pigment epitheliopathy
		Birdshot chorioretinitis
		Multiple evanescent white dot syndrome
	Stromal choroiditis	Vogt-Koyanagi-Harada syndrome
		Sympathetic ophthalmia
	Panuveitis	Behcet’s disease
		Sarcoidosis
		Multifocal choroiditis with panuveitis
	Miscellaneous	Idiopathic panuveitis
		Tubulointerstitial nephritis and uveitis
Infectious	Bacterial	Mycobacterium tuberculosis (serpiginous choroiditis)
	Protozoal	Toxoplasmosis
	Viruses	West Nile virus
		Rubella retinopathy
	Fungi	*Candida albicans*
		*Histoplasma capsulatum* (presumed ocular histoplasma syndrome)
		*Cryptococcus neoformans*
		*Aspergillus fumigatus*
	Helminth	Toxocara
	Others	Endophthalmitis

#### Infectious uveitis

I-CNV can be secondary to either infectious or non-infectious uveitis. Causes of infectious uveitis that result in CNV include histoplasmosis, toxoplasmosis, toxocariasis, tuberculosis, congenital rubella, and West Nile virus [11–16]. Published data on the prevalence of i-CNV secondary to infectious uveitis are scarce, with case series and case reports dominating the literature. Furthermore, prevalence is likely to be geographically specific, with higher rates of presumed ocular histoplasmosis syndrome (POHS) reported in American and European populations and higher rates of toxoplasmosis reported in South Asia [7, 17, 18]. Table 2 summarizes the common clinical features, features of i-CNVs, and associated manifestations as reported in the literature.

**Table 2:** Features of inflammatory choroidal neovascularization (CNV) commonly associated with infectious uveitis

	Presumed Ocular Histoplasmosis Syndrome (POHS) [135–138]	Toxoplasmosis [88, 124, 139, 140]	Intraocular tuberculosis [20, 93, 141, 142]	West Nile virus chorioretinitis [143–145]
Prevalence	Frequent; present in majority cases	Estimated between 0.3–19%	Uncommon; prevalence not known	Only few cases (< 5) reported
Location of CNV	CNV is seen at the edge of a pre-existing scar in the macular or peripapillary region	CNV typically grows close to the edge of an atrophic chorioretinal scar	CNV is typically adjacent to the healed choroidal granuloma or to a healed choroiditis scar	CNV is adjacent to chorioretinal scars
Morphology of CNV	Active lesions have a disciform appearance at the macula, with a green-gray subretinal lacy discoloration and surrounding pigment. Inactive CNV appears as a white disciform scar with fibrovascular tissue	Active CNV appears as an outer retinal lesion close to the scar with associated hemorrhages and intra- or subretinal fluid.	CNV may present as a subretinal lesion with hemorrhages and intra- or subretinal fluid. Rarely, type 1 CNV may be detected only using imaging	CNV presents as a chorioretinal lesion with subretinal fluid and area of retinal hemorrhage
Associated inflammatory lesions	The triad of POHS includes the presence of peripapillary atrophy or pigmentation, histo spots (focal round-shaped chorioretinal lesions), and absence of overlying vitritis	Recurrent disease appears as an oval or circular whitish focal area of retinochoroiditis in the periphery of old atrophic lesions; dense overlying vitritis (headlight-in-fog); perivasculitis with diffuse venous sheathing; segmental arteriolar plaques	Choroiditis may have amoeboid lesions with central healing and active margins (serpiginous choroiditis) or it may present with choroidal granulomas	There is multifocal chorioretinitis with vitritis; multiple active chorioretinal lesions have the appearance of deep, creamy lesions and are 200–1000 μm in size. Inactive lesions are partly atrophic and partly pigmented with a “target-like appearance.”

#### Non-infectious uveitis

The prevalence of CNV secondary to non-infectious uveitis is better defined. In a retrospective multicenter cohort study of 15,137 non-infectious uveitic eyes, Baxter and colleagues report that 2% of patients presenting with posterior or panuveitis had either active CNV or sequelae of past CNV [5]. In contrast, CNV was identified very rarely at the presentation in cases of anterior or intermediate uveitis [5]. Causes of non-infectious uveitis that result in CNV include Vogt-Koyanagi-Harada’s disease, punctate inner choroidopathy, multifocal choroiditis, geographic helicoid peripapillary choroidopathy, and acute posterior multifocal placoid pigment epitheliopathy [1, 18]. Vogt-Koyanagi-Harada disease and punctate inner choroidopathy have been associated with the highest incidence of CNV among these non-infectious uveitides, with adjusted hazard ratios of 3.37 and 8.67 respectively [5]. Various common causes of non-infectious uveitides associated with i-CNV are listed in Table 3 along with their prominent characteristic features.

**Table 3:** Features of inflammatory choroidal neovascularization (CNV) commonly associated with non-infectious uveitis

	Multifocal choroiditis [74, 146, 147]	Punctate inner choroidopathy (PIC) [74, 146]	Serpiginous choroiditis [148–150]	Vogt-Koyanagi-Harada disease (VKH) [151–153]
Prevalence	33–50% cases	76–100% cases	10–25% cases	9–15%
Location of CNV	Associated with inflammatory lesions in the subfoveal or extrafoveal region	Highly focal; associated with inflammatory lesions in the macula	CNV is located near chorioretinal lesions in peripapillary, subfoveal, or extrafoveal areas	Usually extrafoveal; can be subfoveal and associated with chorioretinal scar
Morphology of CNV	CNV appear as subretinal elevations and subretinal fluid with or without associated hemorrhage, closely resembling inflammatory lesions	CNV appear as subretinal elevations and subretinal fluid with or without hemorrhage, closely resembling inflammatory lesions	CNV lesions are deep with associated chorioretinal atrophy, subretinal fibrosis, and pigment clumping	CNV lesions are deep, associated with subretinal or intraretinal fluid, with hemorrhage and exudation.
Associated inflammatory lesions	Multifocal choroiditis present with minimal vitreous inflammation with multiple punched-out, white-yellow lesions (50–200 μm) in the peripapillary, mid-peripheral, and anteriorly to the equator	The lesions are characterized by multiple, small (50–300 μm in diameter), yellow or white, opaque, round lesions scattered throughout the posterior pole, rarely extending to mid-periphery; absence of vitritis	Active lesions appear as gray-white lesions that progress in a geographic manner in the posterior fundus	VKH presents with granulomatous anterior uveitis, posterior synechiae, iris nodules, and stromal atrophy; multiple pockets of subretinal fluid with exudative detachments; sunset glow fundus in the chronic disease.

### Clinical features of inflammatory choroidal neovascularization

#### Signs and symptoms

Classically, i-CNV lesions present when the patient complains of new-onset distortion or metamorphopsia. Sometimes, it may lead to the diminution of vision or a scotoma which is unexplained by the uveitis lesion. Many lesions, especially extrafoveal i-CNVs may be asymptomatic and may be detected only by clinical examination or imaging alone. i-CNVs are usually reported with posterior or panuveitis, as well as in certain cases of intermediate uveitis. Commonly, i-CNVs are diagnosed in a patient on follow-up for uveitis, in which case the CNV lesion may be missed initially due to the presence of associated features such as inflammatory lesions, scars, and pigmentation, as well as intra- or subretinal fluid accumulation [19]. On the contrary, Invernizzi et al. [20] recently demonstrated in three cases that the detection of CNV without associated AMD features (such as drusen) led the authors to perform additional imaging, such as ICGA, which showed the presence of choroidal stromal inflammation leading to the diagnosis of presumed tubercular choroiditis.

i-CNV lesions are usually closely associated with chorioretinal lesions such as scars, lesions, or choroidal granulomas. These lesions can be subfoveal, extrafoveal, or juxtafoveal, and are highly focal. These can be associated with intra- or subretinal hemorrhages and exudation surrounding the lesion. Healed or inactive i-CNVs can result in subretinal yellow-white scars, which may be associated with fibrosis and pigmentation. The presence of intra- or subretinal fluid with i-CNV, as well as serous retinal detachment, may also represent signs of inflammation, leading to a misdiagnosis during cursory examination [1, 10, 19].

#### Contrasting features of i-CNV and neovascular AMD

Patients with AMD-associated CNV have certain differences compared to i-CNVs. Usually, CNVs among patients with AMD can be characterized as type 1 (subretinal), type 2 (outer retinal), or mixed based on clinical and examination (including imaging) findings [21]. A majority of i-CNVs are type 2 lesions with abnormal growth of vasculature into the outer retinal space. AMD CNVs are usually subfoveal and are associated with the presence of drusen and retinal pigment epithelial abnormalities due to the accumulation of lipofuscin material. On the other hand, the retinal pigment epithelium is often intact in individuals with i-CNV [19]. The proposed mechanism of development of i-CNV is the focal breach of the retinal pigment epithelium due to infection/inflammation leading to growth and entry of new vessels into the outer retinal space [19, 22, 23]. Since i-CNV lesions are highly focal, they may respond to fewer therapeutic interventions such as the lesser number of injections compared to AMD patients (as shown by experiences of various authors in Table 4).

**Table 4:** Summary of studies showing the efficacy of anti-vascular endothelial growth factor therapy in inflammatory choroidal neovascularization (studies with sample size ≥ 5 eyes)

Author (year); country	Design; sample size	Disease	Mean no of injections; agent	Mean follow-up	Efficacy outcomes
Roy et al. (2017); India [18]	Retrospective; 30 eyes (28 patients)	Idiopathic choroiditis, toxoplasmosis, panuveitis, VKH, serpiginous choroiditis	2.76; (bevacizumab, ranibizumab)	17.93 ± 14.28 months	Improvement in visual acuity in 53.3%; stabilization in 26.6%
Korol et al. (2017); Ukraine [154]	Prospective cohort; 15 eyes (14 patients)	Toxoplasmosis	1.7 (aflibercept)	12 months	Visual acuity improved from 0.36 to 0.64 (*p* = 0.0002)
Parodi et al. (2014); Italy [76]	Prospective; 7 eyes (7 patients)	Serpiginous choroiditis	1 injection in 12 months (bevacizumab)	12 months	Visual acuity improvement in 52% and stabilization in 57%
Mansour et al. (2012); Lebanon [155]	Retrospective; 8 eyes (8 patients)	VKH, PIC, toxoplasmosis	1.375 (bevacizumab)	5 years	Visual acuity improved (median gain of 3.8 lines)
Iannetti et al. (2013); Italy [156]	Prospective study; 8 eyes (8 patients)	Posterior uveitis	3.75 ± 1.38 (bevacizumab)	19.25 ± 6 months	Visual acuity improved from 0.27 to 0.5 (*p* < 0.05)
Julian et al. (2011); France [157]	Retrospective; 15 eyes (15 patients)	Multifocal choroiditis with panuveitis, ampiginous choroiditis, and others	4.25 (in 12 eyes); 3 eyes received only 1 injection (bevacizumab)	17.6 months	Visual acuity improved from 0.53 to 0.29
Cornish et al. (2011); UK [158]	Retrospective; 9 eyes (9 patients)	PIC	2.34 injections per year (bevacizumab and ranibizumab)	14.9 months	Visual acuity gain was 0.36 LogMAR units
Kramer et al. (2010); Israel [159]	Retrospective; 10 eyes (10 patients)	Multifocal choroiditis, PIC, toxoplasmosis, POHS, serpiginous choroiditis, and panuveitis	2.7 ± 2 (bevacizumab)	13 ± 8 months	Visual acuity improved from 0.87 ± 0.74 to 0.38 ± 0.63 (*p* = 0.005)
Lott et al. (2009); USA [160]	Retrospective; 34 eyes (30 patients)	Multifocal choroiditis, PIC, VKH, idiopathic panuveitis, sarcoidosis, serpiginous choroiditis, toxocariasis, POHS, CMV retinitis, and others	2 (bevacizumab)	7 months	At 6 months, visual acuity improved in 17% and stabilized in 33%
Doctor et al. (2009); USA [161]	Retrospective; 6 eyes (5 patients)	Idiopathic panuveitis, birdshot chorioretinopathy, sympathetic ophthalmia, VKH, and multifocal choroiditis and panuveitis	2.7 (bevacizumab)	15.3 months	Visual acuity improved in 60% of cases
Fine et al. (2009); USA [95]	Retrospective; 6 eyes (5 patients)	Multifocal choroiditis	2.3 (bevacizumab)	6 months	5/6 eyes improved to 20/30 acuity or better at 6 months
Schadlu et al. (2008); USA [87]	Retrospective; 28 eyes (28 patients)	POHS	1.8 (bevacizumab)	22.43 weeks	Visual acuity improved from 0.65 to 0.43 LogMAR units
Adan et al. (2007); Spain [162]	Retrospective; 9 eyes of 9 patients	PIC, serpiginous choroiditis, multifocal choroiditis, POHS, and birdshot chorioretinopathy	7 eyes received 1 injection (bevacizumab)	7.1 months	CNV resolved completely in 100% affected eyes

*CNV* choroidal neovascularization, *CMV* cytomegalovirus, *PIC* punctate inner choroidopathy, *POHS* presumed ocular histoplasmosis syndrome, *VKH* Vogt-Koyanagi-Harada’s disease

Patients with AMD develop CNV due to various contributory factors such as inflammation, complement dysregulation, and growth factor drives such as VEGF drive. Therefore, patients with AMD require constant suppression of VEGF with multiple injections of anti-VEGF agents [24, 25]. In contrast, in patients with i-CNV, with control of inflammation, which primarily drives the development of the CNV, it may be possible that the neovascular drive is extinguished once the inflammation is adequately controlled.

In the context of AMD, various multicenter clinical trials and their extension studies have shown that these patients often require anti-VEGF therapy for several years [26–30]. For instance, the SEVEN-UP study (*n* = 65), a multicenter, non-interventional cohort study, examined the long-term results of patients 7 years after entering the ANCHOR/MARINA trials with monthly anti-VEGF therapy. In this study, 37% eyes maintained visual acuity ≥ 20/70. Sixty-eight percent of study eyes had active exudative disease [27]. In contrast, there are no such long-term reports in patients with uveitis due to i-CNVs.

### Imaging features of inflammatory choroidal neovascularization

#### Fluorescein angiography

Traditionally, fluorescein angiography (FA) has been widely employed in the diagnosis of CNV secondary to various pathologies such as AMD. In AMD, the patterns of CNV include classic (subretinal) or occult CNV (sub-RPE). However, on FA, it may not be possible to characterize the membranes into one of the two categories easily, and therefore, some membranes are referred to as predominantly classic or minimally classic and occult with no classic types [31]. Active CNV lesions of AMD show the leakage of the dye with early hyperfluorescence, which may appear to be from an undetermined source in the occult (type 1) CNVs.

**Fig. 1:**
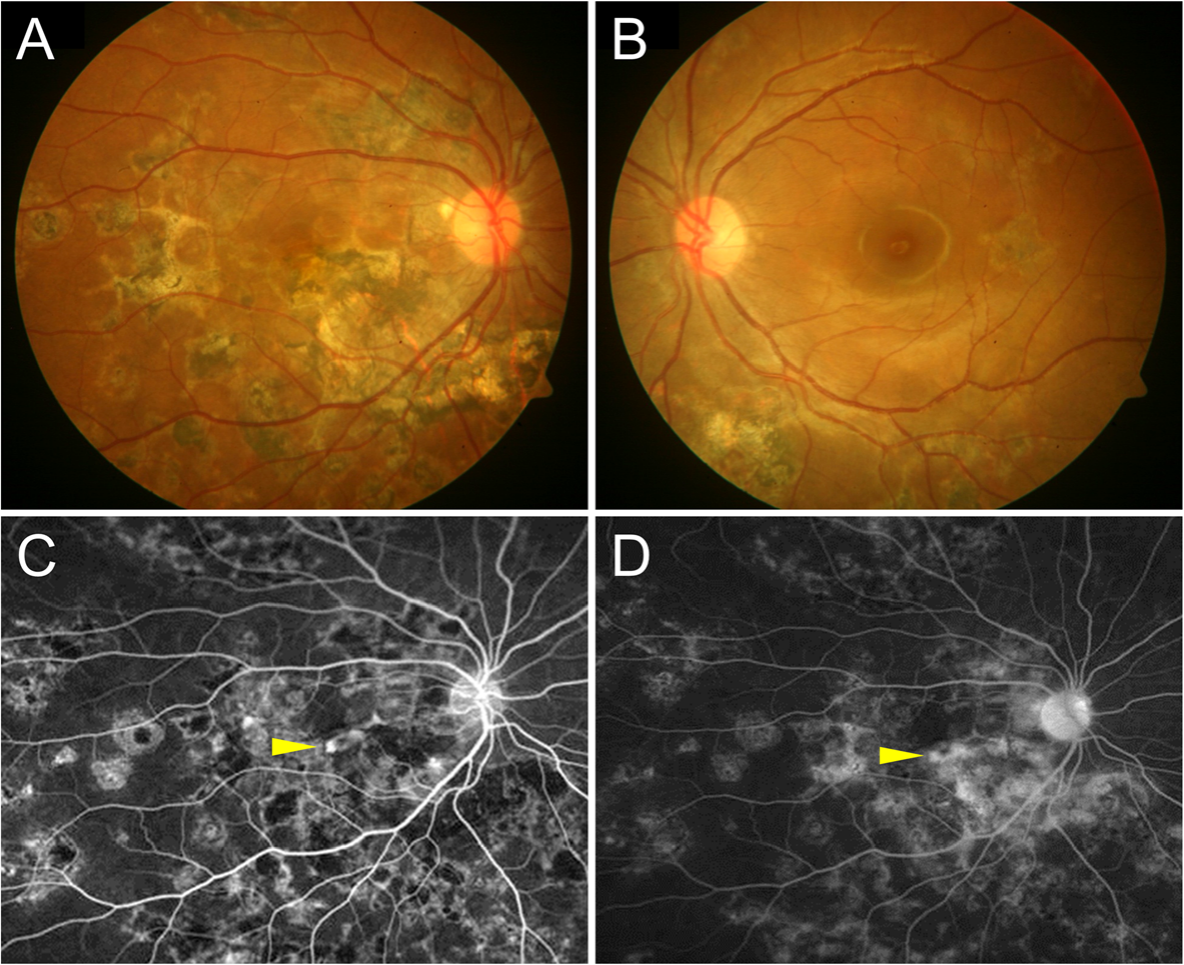
The detection of inflammatory choroidal neovascularization on fluorescein angiography in a patient with tubercular serpiginous-like choroiditis. Fundus photograph of the right eye shows healed choroiditis lesions in the right eye (**a**) and the left eye (**b**). Fluorescein angiography of the right eye shows early hyperfluorescent lesion in the foveal region (yellow arrowhead) (**c**). The late phase angiogram shows progressive hyperfluorescence of the foveal lesion suggestive of choroidal neovascularization (yellow arrowhead) with staining of the healed choroiditis lesions (**d**)

The appearance of CNV in posterior uveitis on FA has not been as well characterized. CNV lesions on FA present with *early iso- or hyperfluorescence* with *late leakage* (Fig. 1) [32]. The presence of surrounding mild hemorrhage causing masking effect on FA may also lead to the diagnosis of CNV. However, the inflammatory retinochoroidal lesions of posterior uveitis may present with markedly similar findings making the differentiation very challenging. Active uveitic chorioretinal lesions show *early isofluorescence (but mostly hypofluorescent) with late leakage*. On the other hand, inactive atrophic lesions show *early hypo-/isofluorescence with late staining* (indicating RPE window defect) without any leakage [1–3]. Thus, there are very subtle differences in the appearance of FA between CNV lesions and active/inactive retinochoroidal inflammatory lesions. In the presence of extensive retinal involvement resulting in scars and pigmentary changes, which may happen in conditions like multifocal choroiditis, serpiginous choroiditis, and VKH disease, the detection of hyperfluorescence due to CNV may be very challenging.

In summary, FA in the detection of CNV in inflammatory conditions may be inconclusive. Additional tests such as OCT and OCTA may be needed to initiate therapy for CNV lesions and a multimodal imaging approach is always recommended.

#### Indocyanine green angiography

**Fig. 2:**
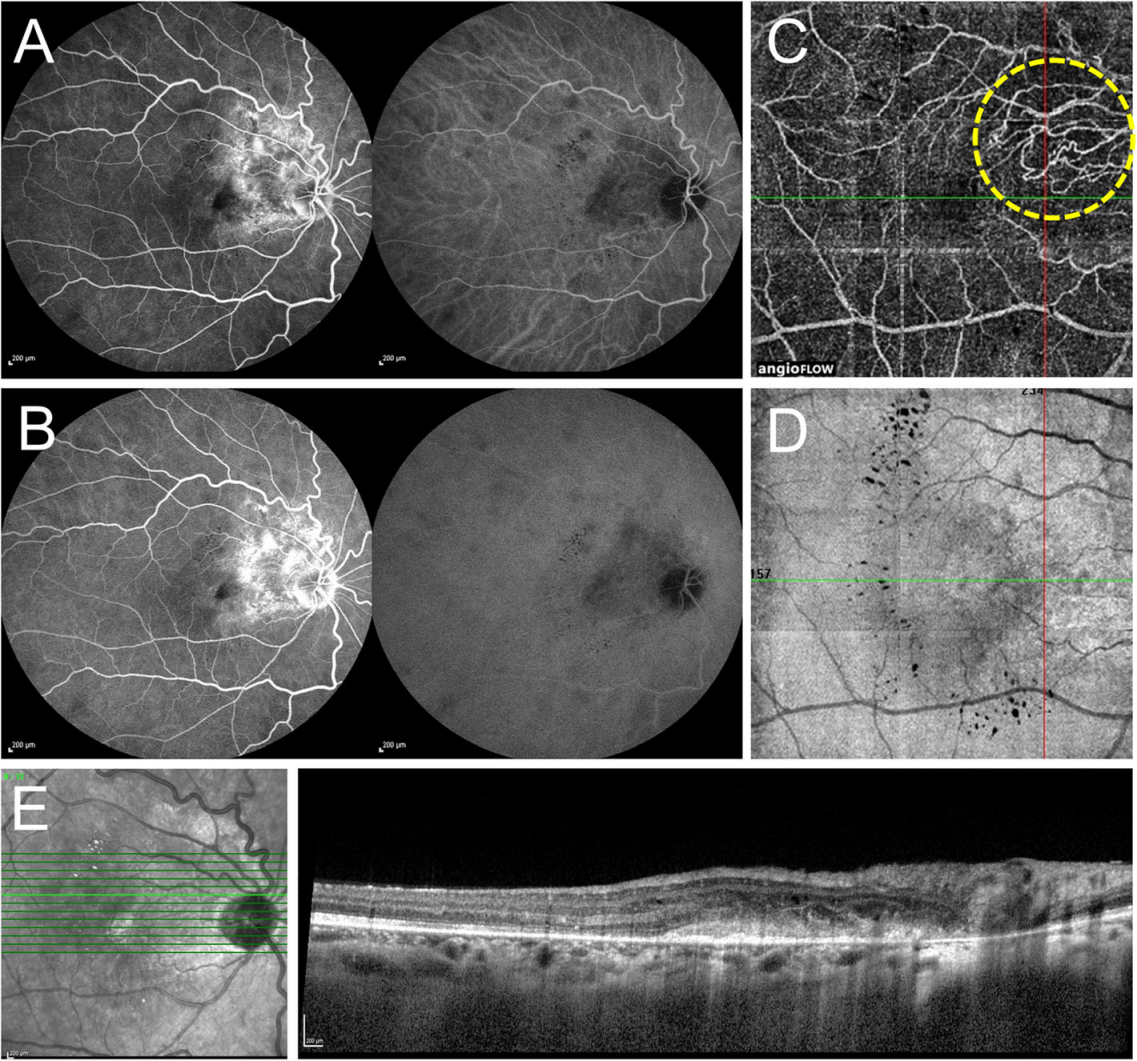
Multimodal imaging in a case of peripapillary inflammatory choroidal neovascularization (CNV) (sea fan type). **a** Combined fluorescein angiography (FA) and indocyanine green angiography (ICGA) show multiple round hypofluorescent lesions in the mid-periphery with early ill-defined hyperfluorescence on FA and area of hypocyanescence with ill-defined choroidal vessels on ICGA temporal to the optic disc. **b** In the late phase FA, the mid-peripheral hypofluorescent lesions become less hypofluorescent/isofluorescent. There is a progressive increase in the hyperfluorescence temporal to the optic disc on FA, suggestive of type 2 choroidal neovascularization. **c** Optical coherence tomography angiography *en face* scan shows the presence of neovascular loops of vessels which have a sea fan configuration although the feeder vessel is not apparent (yellow dashed circle). The scan has been obtained by manually segmenting the image to obtain a slab of 60 μm thickness including the outer retina and choriocapillaris to allow better delineation of the pathology. **d** The corresponding structural *en face* scan does not show any signal loss except in the areas of hard exudates. **e** The optical coherence tomography line scan passing through the area of CNV shows the presence of a hyper-reflective lesion in the outer retina in the peripapillary region suggestive of type 2 CNV associated with retinal thickening and intraretinal cystoid spaces

Indocyanine green (ICG) angiography is an imaging technique that allows a better visualization of the choroid compared to fluorescein angiography [33]. The use of ICG angiography in uveitis permits the identification of choroidal abnormalities such as granulomas, choriocapillaris hypoperfusion, active choroiditis, and hyper-permeability [34]. Neovascular networks composing CNVs are also clearly visualized by ICG, with this technique showing better results in detecting occult lesions compared to fluorescein angiography (Fig. 2) [35].

I-CNVs are mainly represented by classic lesions and are usually clearly visualized by fluorescein angiography [1, 32]. However, a recent study on idiopathic CNVs which share several clinical features with i-CNVs showed that ICG was more accurate than fluorescein angiography in evaluating the size of the neovascular lesions. Furthermore, a reduction of more than 33% in the size of the CNV, measured by ICG angiography 2 months after initiating the treatment, was associated with a favorable outcome [36]. In select cases, ICG angiography can be used to augment the evaluation and follow-up of i-CNVs, identifying possible occult components and allowing these lesions to be differentiated from recurrent inflammatory lesions [37].

In summary, ICG helps to identify both i-CNVs and their associated choroidal alterations in patients with uveitis, thereby allowing a more comprehensive evaluation of the disease.

#### Optical coherence tomography

The introduction of optical coherence tomography (OCT) into clinical practice has profoundly impacted the management of retinal and choroidal diseases. This technique allows clinicians to obtain quasi-histological sections of the ocular structure in a non-invasive and highly repeatable way [38]. For this reason, OCT permits the evaluation of pathological changes of ocular structures and provides a means of assessing responses to treatment. A development of the OCT technique, the “enhanced depth imaging” (EDI) modality, can be used to evaluate choroidal thickness and structural modifications and is particularly useful in the management of uveitis [39].

**Fig. 3:**
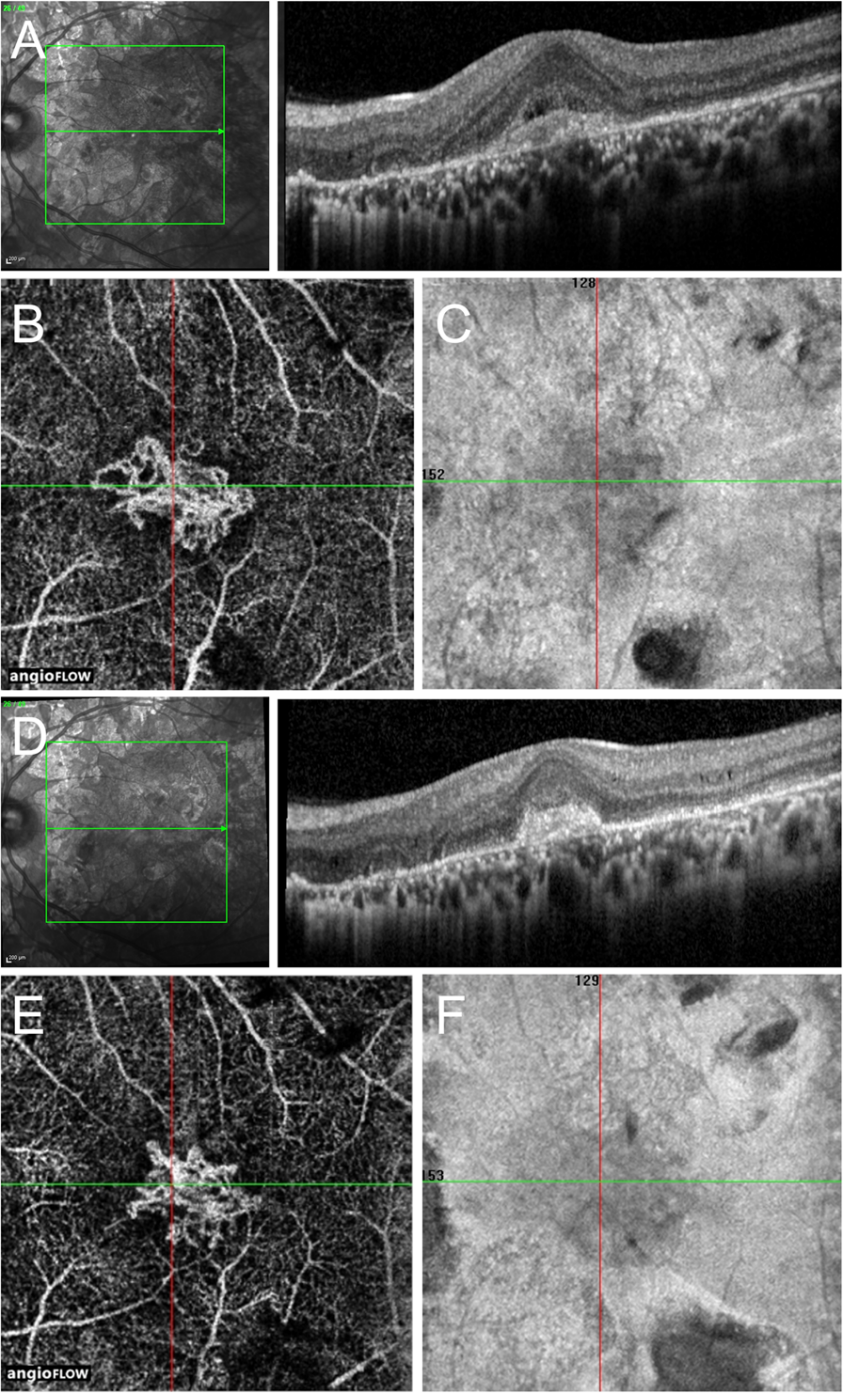
Multimodal imaging of inflammatory choroidal neovascularization (CNV) (medusa head appearance) in a case of multifocal choroiditis. **a** The optical coherence tomography line scan passing through the fovea shows a hyper-reflective lesion in the outer retina with the presence of mild subretinal fluid suggestive of a type 2 CNV. **b**, **c** Optical coherence tomography angiography (OCTA) *en face* 3 × 3 mm scan (along with structural *en face* OCT scan) confirms the presence of a CNV lesion with a medusa head appearance. Similar to Fig. 2, manual segmentation of the scan has been performed to obtain a slab of 60 μm thickness including the outer retina and choriocapillaris to allow better delineation of the CNV. **d** The OCT line scan at 8-month follow-up after one injection of intravitreal ranibizumab shows the persistence of outer retinal hyper-reflectivity but resolution of subretinal fluid. **e**, **f** At this visit, the OCTA *en face* scan (with corresponding structural *en face* scan) shows a decrease in the size of the CNV lesion along with a decrease in vessel caliber and branching

I-CNV usually develops between the retinal pigment epithelium (RPE) and the neurosensory retina, with imaging features comparable to those of classic (type 2) CNVs [1, 18, 40]. On OCT images, these lesions appear as hyper-reflective structures anterior to a disrupted RPE, with solid tissue visualized in the subretinal space (Figs. 2 and 3) [40, 41]. One distinctive OCT feature of i-CNV that helps to distinguish these cases from other type 2 CNVs is the “pitchfork sign.” This sign describes finger-like hyper-reflective projections extending from the CNV area into the outer retinal layers [42]. In a fraction of those cases of CNV as a sequela of uveitis, an occult component of the membrane can be present, appearing as a pigmented epithelial detachment with mixed content [18].

On OCT scans, the activity of CNV is associated with signs of exudation such as retinal thickening, subretinal fluid, intraretinal fluid, intraretinal flecks, and low reflectivity or undefined boundaries of the subretinal material (Fig. 2) [41, 43]. The presence of these features correlate with the leakage on fluorescein angiography and can be used to monitor CNV progression and response to therapy [43]. In a more simplistic approach (and due to the need for quantitative measurements), the central retinal thickness measured on OCT is often used as an objective measure for i-CNV activity [44, 45].

Finally, several uveitides are characterized by the presence of non-neovascular alterations occurring at the level of the RPE, which induce changes in the retina and the choroid. OCT images can help in differentiating such chorioretinal lesions from i-CNVs. In multifocal choroiditis, for instance, acute inflammatory foci are characterized by deeper penetration of the OCT signal underneath the lesion. This sign is usually absent in i-CNV [46, 47]. However, it must be noted that distinguishing CNV lesions from inflammatory non-CNV lesions may be challenging on OCT. Inflammatory chorioretinal lesions may present with very similar features of outer retinal/RPE hyper-reflectivity, intra-retinal edema, sub-RPE fluid, and exudation. Such findings are common in conditions such as MFC, PIC, TB SLC, and other conditions with RPE/choriocapillaris involvement. In such situations, a combination of imaging tools such as FA and ICGA, as well as OCTA, may be useful in determining the characteristics of the lesions [48].

#### Optical coherence tomography angiography

##### Principle and algorithms of optical coherence tomography angiography

The development of OCTA has revolutionized the study of pathogenesis, staging, diagnosis, and management of CNV lesions in various ocular conditions, especially AMD. OCTA helps in the reconstruction of retinochoroidal microvascular network by using endoluminal flow as contrast non-invasively [49]. The development of OCTA has added a new dimension of functional vascular network imaging to the existing capabilities of OCT allowing it to image end-arterial system without the need of a dye injection [50]. The OCTA devices commercially available consist of various image processing algorithms. The most commonly used algorithms include the following: (1) complex signal-based OCTs, such optical microangiography (OMAG) (Zeiss Angioplex ®, Carl Zeiss Meditec Inc., Dublin, CA); (2) amplitude-based OCT signal, e.g., amplitude decorrelation (Optovue AngioVue ®, Optovue, Inc., Fremont, CA); and (3) speckle and phase variance. Each algorithm has its own advantages and limitations [51, 52]. With advancing technology, there have been significant improvements in the OCTA processing software. It is expected that new algorithms that combine merits of various methods will become available to achieve better image quality in the future. Presently, none of the available algorithms are perfectly designed to identify the retinochoroidal layers by automatic segmentation in all the patients without any errors. In the context of uveitis, automatic segmentation is even more challenging due to the abnormalities involving the chorioretinal layers resulting from scarring, choroiditis, intraretinal/subretinal fluid, and other pigmentary changes [53, 54]. Therefore, caution must be exercised while interpreting the OCTA images in the clinics. It is best to manually correct the segmentation and assess the structural en face images to avoid false interpretation due to artifacts such as shadowing and projection artifacts, among others.

To overcome the inherent limitations of OCTA, modern techniques such as machine learning algorithms can enable more sophisticated image analyses and interpretation. For instance, machine learning algorithms can allow identification of CNV by automated segmentation, improving the accuracy of detection, permitting quantification, and delineating the boundaries of the lesion [55]. In the future, such techniques may be employed in the diagnosis of i-CNV.

##### Utility of optical coherence tomography angiography in age-related macular degeneration

Because of its utility, OCTA is very useful in the assessment of neovascular networks in neovascular AMD. Jia et al. first reported that OCTA was able to detect and quantify CNV in five eyes with AMD [56]. In a study by De Carlo et al., the sensitivity of 50% (4 of 8) and specificity of 91% (20 of 22) was observed when OCTA was compared to FA in their ability to detect CNV [57]. This relatively low sensitivity was attributed to the small sample size and presence of large retinal hemorrhages. OCTA is capable of detecting type 1, type 2, and type 3 CNV lesions in AMD [58, 59]. Various studies have compared the sensitivity and specificity of OCTA with conventional dye-based angiographies in detecting CNV. The sensitivities range from 50 to 87%, and specificities range from 91 to 100% [60–64].

OCTA is also useful in determining the characteristic microvascular details of the CNV complex in AMD. This allows detailed analyses such as quantification of the area, evaluation of the branching pattern, and detection of novel measures of CNV activity such as fractal analysis [65, 66]. Morphological pattern analysis of CNV on OCTA has allowed identification of two major categories: (1) sea fan configuration with densely packed networks and capillary sprouts; and (2) medusa head pattern with large diameter vessels sprouting from a central vessel. OCTA has been shown to be useful in the determination of biological markers of activity of the CNV lesion, i.e., the presence of features such as perilesional halo, increased complexity of branching and capillary sprouts, and alteration of choriocapillaris flow at the margins are indicators of an active lesion [66, 67]. On the other hand, lesions with large trunk vessels with minimal branching indicate residual inactive networks in AMD which may not require therapy.

In AMD, OCTA has enabled identification of CNV lesions that are non-exudative and asymptomatic, i.e., treatment-naïve nascent lesions which can progress to exudation and development of larger CNV lesions [68, 69]. Thus, OCTA can permit early identification of these CNV lesions in AMD, potentially enabling the clinician to initiate early therapy. Lastly, OCTA also provides documentation of vascular remodeling and aids in the understanding of the biological behavior of CNV networks in AMD [70]. This can provide insights into the pathogenesis of the disease.

In summary, OCTA has found innumerable applications in the assessment of CNV in AMD over the past few years. The technology of OCTA in AMD is still evolving and holds promise in improving the outcomes in these patients.

##### Optical coherence tomography angiography in the detection of inflammatory choroidal neovascularization

Compared to neovascular AMD, the applicability, usefulness, and limitations of OCTA in the evaluation of i-CNV need to be explored in various areas. Similar to neovascular AMD, OCTA can allow precise delineation of i-CNV lesions in patients with uveitis. A number of authors have shown the utility of OCTA as a non-invasive modality in the detection of CNV.

In a study by Cheng et al., the ability to detect CNV and differentiate it from the inflammatory lesions on OCTA was assessed. In this series, 26 patients with a diagnosis of multifocal choroiditis were evaluated using OCTA and conventional FA [71]. Using FA, active type 2 CNV networks were identified in all the eyes. These findings were also corroborated using OCTA. On OCTA, three eyes had severe motion artifacts limiting the findings. The authors concluded that OCTA has an advantage of differentiating i-CNV lesions from inflammatory lesions as these do not show any blood flow signals [71]. Similarly, in a recent series by Zahid et al. [72], neovascular flow signals were evaluated using OCTA in 14 patients with multifocal choroiditis. Yee et al. demonstrated that OCTA allows non-invasive diagnostic imaging of i-CNV and its follow-up after therapy [73]. Thus, there are various case reports in which OCTA has been used to detect neovascular flow lesions. OCTA allows non-invasive i-CNV detection in punctate inner choroidopathy [74], multifocal choroiditis [46] and other white dot syndromes [75], serpiginous choroiditis [76], cat-scratch disease [77], and TB-associated choroiditis [73]. In most of these published studies, OCTA has been used to confirm the presence of i-CNV networks in uveitis and demonstrate the neovascular regression following therapeutic intervention.

As mentioned earlier in the study by Cheng et al. [71], OCTA particularly has an advantage over FA in distinguishing neovascular lesions from inflammatory lesions. In a series of 13 patients (18 eyes) by Astroz et al. [48], patients with multifocal choroiditis were evaluated using various imaging tools to characterize inflammatory and i-CNV lesions. The authors showed in their results that on OCTA, i-CNV showed abnormal blood flow in almost all eyes and also in two lesions previously diagnosed as inflammatory lesions. Compared to other imaging techniques, OCTA allowed a diagnosis of i-CNV in additional 14% cases misdiagnosed as inflammatory lesions on other tools such as FA and OCT [48].

Certain published case reports have demonstrated distinct advantages of OCTA over conventional FA and OCT in the detection of i-CNV, especially when the FA and OCT are inconclusive. In a case report, Nozaki et al. evaluated a patient of multiple evanescent white dot syndrome (MEWDS) and i-CNV using conventional FA, OCT, and OCTA [75]. The authors observed that FA showed significant leakage and pooling of dye due to macular edema and serous retinal detachment. Similarly, the OCT was inconclusive. It was only through OCTA that the authors were able to observe a neovascular complex and diagnose the presence of an i-CNV [75]. Similarly, Baumal et al. demonstrated in a young female patient with multifocal choroiditis that OCTA can be useful in confirming the presence of an i-CNV when the conventional dye-based FA and SD-OCT was inconclusive [78]. Levinson studied a larger cohort of 12 patients with PIC and multifocal choroiditis of which 17 eyes had suspected i-CNV lesions. In this study, the authors were able to identify i-CNV in 15 eyes of 11 patients as a neovascular network in choriocapillaris/outer retinal layer. Among the seven eyes that underwent FA imaging, i-CNV was detected in only four eyes using FA as an abnormal staining (but no clear-cut vessels were visible) [74]. Another case report by Nakao et al. of a patient with PIC showed the superiority of OCTA in detecting i-CNV where FA showed only staining and possible leakage but was inconclusive [79].

Aggarwal et al. recently studied OCTA features of TB-associated choroiditis and compared the findings to conventional imaging including FA, ICGA, and OCT. The study showed that OCTA, for the first time, was able to identify type 1 neovascular networks in TB choroiditis [80]. In this study, the authors showed that findings were inconclusive on FA with only an ill-defined hyperfluorescence in the late phase. ICGA also showed only subtle ill-defined hyper-cyanescence that did not confirm the presence of i-CNV. OCT showed only low-lying pigment epithelial detachments but no intra- or subretinal fluid. Thus, the manuscript concluded that without OCTA, it is impossible to rule out neovascular networks when FA, ICGA, and OCT are inconclusive [80]. In addition, the presence of associated pathologies such as chorioretinal scarring, choroiditis, and chorioretinal lesions may make it difficult to identify i-CNV among patients with posterior uveitis [3]. Thus, the use of OCTA provides objective evidence of the presence of CNV lesions and greatly aids in the decision-making for the use of anti-VEGF agents (Figs. 2 and 3).

##### Future directions of optical coherence tomography angiography

Studies with larger sample sizes and prospective methodologies are needed to determine the benefits of OCTA over conventional imaging such as FA, ICGA, and OCT in terms of sensitivity and specificity. Unlike in AMD, quantitative analysis of i-CNV lesions (such as area analysis, lesion size, vessel density analysis, and fractal dimension analysis) have not been performed thus far. Further studies are required that enable better correlation of FA, ICGA, and OCT with OCTA especially in missed cases of i-CNV so that the detection and treatment rates can be improved. While evidence does show that OCTA may have several advantages over conventional imaging, its disadvantages such as motion artifacts, inaccurate segmentation, and projection artifacts need to be addressed. Studies that determine whether OCTA is able to detect i-CNV lesions early and result in better visual outcomes need to be planned in the future.

#### Near-infrared autofluorescence imaging

The technique of near-infrared autofluorescence (NIR AF) using confocal scanning laser ophthalmoscope (SLO) system has been employed in the study of various retinal diseases. NIR AF originates from the melanin of the RPE and has applicability in various conditions such as AMD and CNV due to various causes [81–85]. Among patients with neovascular AMD, NIR AF often shows signal blockade due to subretinal hemorrhage due to CNV. NIR AF signal may be decreased in areas of exudation around the CNV. NIR AF also allows adequate visualization of associated drusen in AMD CNV [85]. On the contrary, i-CNVs may show a different pattern on NIR AF imaging, potentially allowing differentiation between inflammatory lesions and neovascular membranes. NIR AF of active lesions may show increased AF signal, whereas the i-CNV may appear black/grayish with patchy AF signal [71]. Future studies are needed to further characterize the appearance of inflammatory and i-CNV lesions on NIR AF and explore whether this tool can be used to differentiate between CNV related to AMD and other causes.

### Management of inflammatory choroidal neovascularization

#### Anti-vascular endothelial growth factor therapy

##### Mechanism of action

The development of anti-angiogenic therapies based on the current understanding of the molecular events in CNV has helped overcome a barrier the management of CNV. Anti-VEGF drugs pharmacologically target two crucial pathological changes induced by VEGF: permeability and angiogenesis.

##### Anti-VEGF therapy for AMD

Large multicenter randomized controlled trials such as MARINA, ANCHOR, and others have conclusively established the role of serial anti-VEGF injections in AMD [28, 29, 86]. Anti-VEGF therapy is, therefore, the treatment of choice for CNV secondary to AMD. Multiple studies have also demonstrated the utility of anti-VEGF treatment in the management of CNV secondary to other inflammatory etiologies. It is imperative to note however that unlike AMD, in i-CNV, there are no randomized controlled trials (with double masking and comparison to sham/alternate therapies) testing the efficacy of anti-VEGF agents in the resolution of the CNV or improving visual acuity. There are several reasons for this, including the rarity of the CNV occurrence and heterogenous patient profile in terms of disease manifestations, need for concomitant anti-inflammatory therapy, and challenges in the diagnosis.

##### Efficacy of anti-VEGF in i-CNV

Anti-VEGF therapies have been used for the management of i-CNVs associated with both infectious and non-infectious uveitic entities. There are several case reports and case series documenting successful resolution of i-CNV following anti-VEGF therapy, with or without concomitant anti-inflammatory (either corticosteroid and/or immunosuppressive therapy). Table 4 provides details of various relevant studies on the efficacy of anti-VEGF therapy for i-CNV.

Role in infectious uveitis

Among patients with POHS, intravitreal injections of anti-VEGF agents have been successfully used to treat CNV. A retrospective case series of 28 patients presenting either juxta- or subfoveal CNV related to POHS has demonstrated that intravitreal bevacizumab leads to visual acuity improvement in 71% eyes and a stabilization in 14% of cases over an average follow-up of 22 weeks [87]. Similarly, in ocular toxoplasmosis, Ben Yahia et al. [88] have reported the effective use of bevacizumab in the successful treatment of CNV due to ocular toxoplasmosis in two patients. Shah et al. [89] reported clinical improvement in the treatment of CNV secondary to ocular toxoplasmosis with a single administration of ranibizumab in a patient. A few cases of CNV associated with ocular toxocariasis have been managed with anti-VEGF agents. Lyall et al. [90] reported a case of ocular toxocariasis with CNV treated with intravitreal injection of ranibizumab. Yoon et al. [91] have reported a similar case of toxocariasis with CNV treated with intravitreal ranibizumab and bevacizumab injections combined with oral albendazole. Julian et al. [92] conducted a study involving 15 patients diagnosed with CNV secondary to uveitis, including one patient with tuberculous uveitis. All the patients received intravitreal bevacizumab injections for the treatment of CNV lesions. At 17-month follow-up, nearly 80% of eyes showed significant improvement in visual acuity and macular thickness after a mean 4.25 injections. Kim et al. [93] reported a case series of patients with active CNV as a result of tuberculous chorioretinitis. The patients had a notable improvement in their clinical course with the retention of their baseline visual acuity.

Role in non-infectious uveitis

Anti-VEGF therapies are also very efficacious in the management of CNV associated with non-infectious uveitis. Wu et al. [94] reported successful outcomes of intravitreal injections of bevacizumab for CNV associated with VKH disease in two patients. Fine et al. [95] have reported the outcome of bevacizumab and ranibizumab intravitreal injections in 6 eyes affected by CNV secondary to multifocal choroiditis. The results at the end of follow-up (mean follow-up 42 weeks, range 25–69 weeks) showed an improvement of visual acuity values better than 20/30 and reduced activity of CNV lesion in 5 eyes. Another retrospective study analyzed the efficacy of bevacizumab administered for CNV not related to age-related macular degeneration. This study included 12 eyes affected by multifocal choroiditis [96]. Similarly, a recent case report has described a favorable outcome after treatment of CNV associated with serpiginous choroiditis with intravitreal injection of ranibizumab [97]. While CNV is rarely associated with acute posterior multifocal placoid pigment epitheliopathy (APMPPE), a single case report recently describes a 14-year-old girl who developed CNV associated who was effectively treated with a single intravitreal ranibizumab injection with improvement in visual acuity from 20/40 to 20/20 through a follow-up of 12 months along with the stabilization of the CNV [98].

Thus, anti-VEGF agents form the first-line therapy for CNV lesions associated with ocular inflammation and have been used to treat active CNV lesions in various infectious as well as non-infectious uveitic entities.

#### Corticosteroid therapy

##### Mechanism of action

Corticosteroids have been used for decades and still represent the commonest choice in the treatment of uveitis due to their strong and rapid anti-inflammatory effects [99]. In addition to the inhibition of pro-inflammatory factors, transcription and the suppression of prostaglandin and interleukin synthesis, corticosteroids interfere with the effects of VEGF [100, 101]. Reducing the VEGF stimulus to the growth of new vessels and decreasing inflammation, the primary cause for VEGF release, these drugs remain a valuable option for the treatment of i-CNVs.

##### Role of oral corticosteroids

Before anti-VEGF agents became available, corticosteroid therapy was the only medical option for the treatment of CNVs [102]. Patients were usually treated with a course of 1 mg/kg/day of oral corticosteroid for approximately 7 days followed by slow tapering. With this approach, several authors reported stabilization of visual acuity in more than 80% of the patients affected by subfoveal i-CNVs [2, 102]. High-dose systemic corticosteroids can control ocular inflammation as well as the stabilization of i-CNV lesions in patients with posterior uveitis.

##### Local and intravitreal corticosteroids

High-dose systemic corticosteroids are limited by their adverse effect profile. Martidis et al. conducted a study comparing high-dose (1 mg/kg) oral prednisolone and a single sub-Tenon injection of triamcinolone acetonide for subfoveal i-CNV due to POHS [102]. In this study, oral prednisone resulted in a short-term improvement in visual acuity, which stabilized over longer follow-up. Sub-Tenon’s triamcinolone group achieved similar final stabilization without the initial improvement. Rechtman et al. also evaluated the use of intravitreal triamcinolone acetonide in ten patients with POHS [103]. Thirty percent patients showed improvement, whereas 50% showed stabilization in visual acuity. Intravitreal dexamethasone implant (Ozurdex®) has not been employed in the management of i-CNV yet.

Combination therapies with corticosteroids

Local [104] or systemic [105, 106] steroid treatment have been combined with photodynamic therapy (PDT), allowing for a decrease in the number of PDT sessions and, for the first time, to achieve an improvement in visual acuity. Intravitreal corticosteroids (triamcinolone acetonide) has been combined with bevacizumab for recurrent i-CNV in VKH disease in a case report by Pai et al. [107] The authors demonstrated complete resolution of i-CNV and ocular inflammation after combined therapy and systemic steroids until 1 year after follow-up.

When VEGF agents demonstrated favorable results in patients affected by i-CNV (both in terms of visual outcomes and their side-effect profile) [45], the use of corticosteroids alone or associated with PDT for the treatment of this condition has gradually decreased. Despite this, with inflammation being the primary trigger for VEGF increase and consequent CNV development in uveitis, corticosteroids are still considered a valuable option in the management of these conditions.

##### Limitations of the use of corticosteroids

There are currently no data available from randomized controlled clinical trials comparing the efficacy of anti-VEGF agents alone versus corticosteroids alone or as a combined therapy. A proper guideline on the use of corticosteroids in i-CNVs is conspicuously absent. In the face of uncertainty, the wisest approach seems to be to control the inflammatory stimulus by the use of systemic steroids [1] while simultaneously treating the neovascular component with intravitreal injections of anti-VEGFs [19].

#### Immunosuppressive therapy

##### Pre-clinical evidence and mechanism of action

Various corticosteroid-sparing immunosuppressive agents have been used as an off-label therapy in patients with uveitis. As discussed in the preceding sections, most cases with uveitis and i-CNVs are managed using systemic/local corticosteroids and anti-VEGF injections. However, there are situations where corticosteroids may be relatively contraindicated, such as patients with impaired steroid responsiveness or patients with a history of severe systemic/ocular corticosteroid-related adverse events. Immunosuppressive agents, by the virtue of their anti-inflammatory action, can curb angiogenesis and therefore limit the development of CNV.

In an experimental study of laser-induced CNV in mice, the role of tumor necrosis factor (TNF)-α receptors was evaluated [108]. CNV was induced in TNF-α receptor 1a and 1b (−/−) mice and the expression of TNF-α in the RPE, and the choroid was determined using western blot analysis. In this experiment, it was observed that TNF-α levels were elevated in the chorioretinal tissue in mice with CNV. The absence of TNF-α receptors increased endothelial cell apoptosis and led to a reduced inflammatory cellular response [108]. This experiment provides a preclinical evidence of the use of anti-TNF-α agents in the management of i-CNV. In a murine model of laser-induced i-CNVs, the anti-TNF-α agents (etanercept and infliximab) were shown to be effective in decreasing the CNV size and leakage on fluorescein angiography compared to the control group [109]. These experiments support the role of immunosuppressive agents such as TNF-α in the management of i-CNVs. At optimal doses, intravitreal injection of infliximab (an anti-TNF-α agent) in experimentally induced CNV has been demonstrated to reduce the area of CNV and decrease the levels of VEGF (by ELISA and immunofluorescence testing) without any cytotoxic effects on the RPE [110].

##### Systemic immunosuppressive therapy

Systemic immunosuppressive therapy can result in resolution of i-CNV. A case report published in 1998 by Kilmartin et al. demonstrates a case of a 3-year-old boy with sympathetic ophthalmia who developed i-CNV resulting in the worsening of visual acuity. Systemic cyclosporin resulted in the stabilization of the lesion with resolution of associated edema and hemorrhage [111]. In a series by Dees et al., 14 patients (17 eyes) with i-CNV and posterior uveitis were enrolled, of which 3 had extrafoveal CNV, 6 had juxtafoveal CNV, and 8 had subfoveal CNV. Of the 11 patients that received systemic immunosuppressive therapy with agents such as cyclosporin, CNV resolved in most eyes [112]. Neri et al. performed a prospective open-label interventional study evaluating the efficacy of systemic corticosteroids and mycophenolate mofetil for controlling juxta/sub-foveal i-CNV, unresponsive to traditional immunosuppressive therapy. Of the 12 eyes (9 patients), all patients showed stable or reduced lesion size at 12 months [113]. Ganesh et al. studied a large series of 49 eyes (43 patients) with i-CNV. The authors managed 17 eyes with systemic immunosuppressive therapy with favorable results [114].

Immunosuppressive therapy may also be combined with other forms of therapy such as PDT. In a retrospective study by Hogan et al., of the 6 patients, 2 with subfoveal i-CNV were treated with systemic immunosuppression (oral mycophenolate mofetil). This approach was effective in decreasing the fluorescein angiographic leakage from i-CNVs. The authors showed that combination PDT and systemic immunosuppression was a useful therapeutic option [115]. Systemic methotrexate has also been used in combination with intravitreal ranibizumab in a patient with VKH disease complicated by i-CNV [116].

##### Intravitreal immunosuppressive agents

Intravitreal injections of immunosuppressive agents may also be employed in the management of i-CNV. Agents such as intravitreal methotrexate may have a role in the management of i-CNV as shown by Mateo-Montoya et al. In this manuscript, the authors describe a case of a 25-year-old lady with multifocal choroiditis with i-CNV who had received three prior injections of intravitreal ranibizumab. The authors treated this patient with intravitreal methotrexate (single injection) with improvement in visual acuity and no recurrence of CNV lesion at 20 months follow-up [117]. While there are very few such published reports, we have had success in treating i-CNV with intravitreal methotrexate, in patients who either cannot tolerate or afford other forms of therapy.

##### Advantages and limitations of the use of immunosuppressive agents

Knowledge of the use of immunosuppressive agents for i-CNVs is increasing with passing time. However, there are no clear guidelines on the use of these agents, nor on the choice of agents for managing i-CNVs. The exact mechanisms by which immunosuppressive therapies act on the CNV is not yet clear. Therefore, further studies that evaluate the efficacy of immunosuppressive therapies (systemic and/or local) that highlight the choice of the agent, timing and number of injections, and outcome measures are necessary.

Since there are no guidelines on the use of anti-VEGF, corticosteroid or immunosuppressive therapies for i-CNV, a randomized clinical trial is necessary. In the context of uveitic macular edema, where a similar dilemma exists, the National Eye Institute (NEI) is currently recruiting patients in a multicenter clinical trial, Macular Edema Ranibizumab versus Intravitreal Anti-inflammatory Therapy Trial (MERIT), to evaluate the relative efficacy and safety of intravitreal methotrexate, intravitreal ranibizumab, and the intravitreal dexamethasone implant [118]. A similar approach is needed for the management of i-CNVs.

#### Other therapeutic strategies

Other various therapeutic strategies have been used in the literature in the management of i-CNVs. These have been summarized in the following sections.

##### Photodynamic therapy

As mentioned in the preceding sections, PDT has been variably used in the management of CNV associated with uveitis, often combining it with anti-VEGF therapy or corticosteroids. The consensus of the use of PDT alone is that it can stabilize but can rarely improve the visual acuity. PDT with verteporfin has been used in the monotherapy of CNV associated with serpiginous choroiditis, POHS, and PIC [119–121]. In a prospective pilot study of 19 patients with i-CNV, standard PDT with verteporfin was performed for patients with diagnoses of PIC, POHS, and multifocal choroiditis. The authors suggested that PDT may perform better in CNV due to ocular inflammation compared to AMD over a period of 1 year [122]. However, the exact reasons for this is not clear.

Long-term results of PDT have been extensively studied in CNV lesions occurring in association with toxoplasma retinochoroiditis. In a study of 8 patients, classic or predominantly classic CNV was treated using PDT. Persistent CNV regression was achieved in all the patients at 2 years [123]. Neri et al. evaluated the long-term (4-year) outcome of PDT in CNV lesions associated with toxoplasmosis in 9 patients. The authors observed stable/improved visual acuity and stabilized CNV diameters after PDT at 4 years’ follow-up [124]. In other case reports, PDT has been combined with either bevacizumab or with intravitreal triamcinolone acetonide in the treatment of CNV associated with toxoplasmosis [125, 126].

PDT has also been employed in the management of CNV lesions associated with other pathologies such as VKH disease [127, 128]. Nowilaty et al. performed PDT in six eyes of 6 patients with VKH disease who developed CNV. Three eyes showed the development of submacular fibrosis. All the eyes showed stabilization of visual acuity and CNV lesions [127]. However, in another case report, retinal pigment epithelial alterations raised concerns following PDT for CNV associated with VKH disease [128].

Among patients with multifocal choroiditis, PDT has been performed either as a monotherapy [129] or in combination with immunosuppression [115] or anti-VEGF agents [130]. In all these series, PDT was associated with stabilization of CNV and visual acuity. In summary, PDT is rarely used as monotherapy for managing i-CNV in the present times.

##### Surgical excision

Surgical excision of CNV lesions was a historical treatment modality that has gone out of favor in the modern era. This treatment option was selected by ophthalmologists when the sole alternative therapy was laser photocoagulation, which often resulted in visual loss. In a series of 43 eyes of 40 patients with CNV not to related AMD by Benson et al., surgical excision of the subfoveal CNV lesions was performed. In this series, 79% patients showed either improved or unchanged visual acuity following surgery. Recurrence of CNV was noted in 23% eyes for whom repeat surgery was performed [131]. CNV lesions in toxoplasma retinochoroiditis [132] and candida endophthalmitis [133, 134] have been also treated using submacular surgery.

In the era of anti-VEGF therapy, submacular surgery has not found popularity. Thus, this treatment option is no longer preferred and very rarely, if ever, employed in present-day clinical practice.

## Conclusions

The detection of CNV is challenging in patients with uveitis due to the difficulties of visualizing the lesion amidst choroiditis lesions, scarring, and pigmentation. Based on various case reports and series evaluating patients of posterior uveitis with suspected i-CNV, it can be concluded that OCTA in conjunction with FA, ICGA, and OCT can help in improved detection of CNV lesions, especially in cases where conventional imaging is inconclusive. OCTA can also be used to non-invasively follow-up such lesions. Importantly, there should be a high index of suspicion for identifying neovascular flow lesions that may be considered to be inflammatory lesions on examination and conventional imaging. Interpretation of OCTA requires careful review of the images to exclude any image artifacts and incorrect segmentation errors. Since the current OCTA are first generation devices, further improvement in technology may advance our imaging capabilities. As shown in various reports, OCTA is a very useful modality in the diagnosis and follow-up of i-CNV lesions, and further studies may help evaluate its role in determining the endpoint for treatment.

FA and ICGA, the two gold standard dye-based angiographic techniques, provide significant information regarding the retinochoroidal pathology in uveitis, including the level and severity of inflammation, presence of focal lesions, and vascular changes including neovascularization. OCTA appears to provide certain advantages over these existing tools in the detection of neovascular flow lesions in uveitis but certainly does not replace the information provided by the other imaging tools in the present times. Thus, while OCTA is a promising and exciting tool, significant advancements in the technology are needed to establish its role in the practice of uveitis and ocular inflammation.

Many treatment modalities are available in the management of i-CNV associated with uveitis. The general principle of treatment is to limit the inflammatory response with corticosteroids and/or immunosuppressive agents. If the inflammation is unilateral, local therapies such as intravitreal dexamethasone implant, methotrexate, or triamcinolone acetonide can be considered, which can also help to reduce the size of the CNV lesion. Anti-VEGF agents are highly efficacious and are usually employed as the first-line agents for treating CNV associated with uveitis, keeping in mind that the inflammation needs to be controlled for the best outcome and reduction of recurrences. PDT is uncommonly used in the anti-VEGF era due to its limitations in improving visual acuity and potential adverse effects. In the future, novel anti-inflammatory agents and immunosuppressive agents, including intravitreal injections, may become available for the management of i-CNV.

## Abbreviations

AMD: Age-related macular degeneration
APMPPE: Acute posterior multifocal placoid pigment epitheliopathy
CNV: Choroidal neovascularization
EDI: Enhanced depth imaging
FA: Fluorescein angiography
ICG: Indocyanine green
KP: Keratic precipitates
MSC: Multifocal serpiginoid choroiditis
OCT: Optical coherence tomography
OCTA: Optical coherence tomography angiography
PDT: Photodynamic therapy
PIC: Punctate inner choroidopathy
POHS: Presumed ocular histoplasmosis syndrome
RPE: Retinal pigment epithelium
TB: Tuberculosis
TNF: Tumor necrosis factor
VEGF: Vascular endothelial growth factor
VKH: Vogt-Koyanagi-Harada
WNV: West Nile virus
